# Supplementary material for: Efficacy of simultaneous infusion of tirofiban with intravenous thrombolysis in patients with acute anterior choroidal or paramedian pontine infarction: TITACIPPI study
Source: Front Neurol. 2026 Jan 22;16:1692576. doi: 10.3389/fneur.2025.1692576 (PMC12872556; doi:10.3389/fneur.2025.1692576)
Supplement: Supplementary file 1 [file Presentation_1.PPTX]

## Slide 1
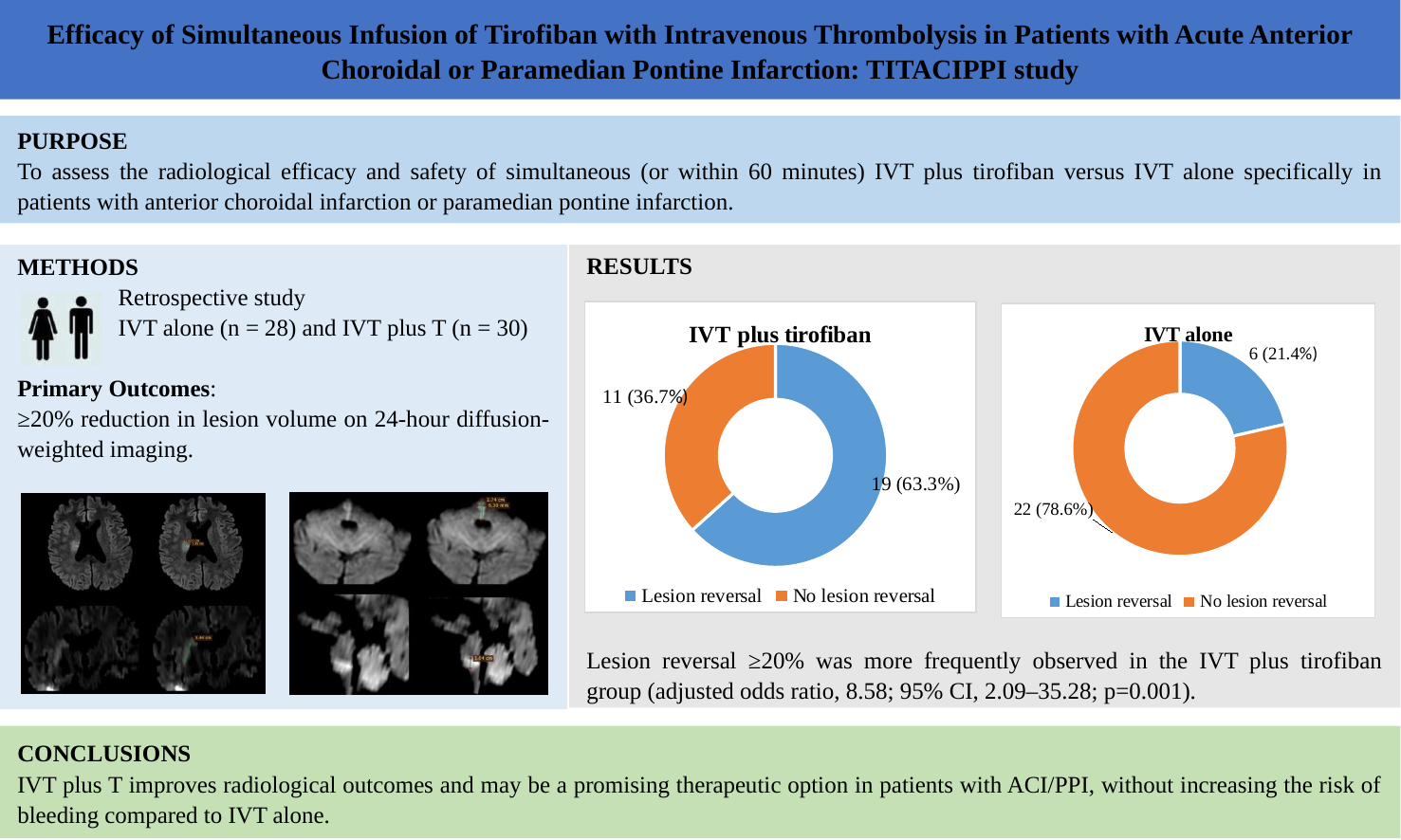

Efficacy of Simultaneous Infusion of Tirofiban with Intravenous Thrombolysis in Patients with Acute Anterior Choroidal or Paramedian Pontine Infarction: TITACIPPI study
PURPOSE
To assess the radiological efficacy and safety of simultaneous (or within 60 minutes) IVT plus tirofiban versus IVT alone specifically in patients with anterior choroidal infarction or paramedian pontine infarction.
METHODS
 Retrospective study
 IVT alone (n = 28) and IVT plus T (n = 30)
Primary Outcomes:
≥20% reduction in lesion volume on 24-hour diffusion-weighted imaging.
RESULTS
Lesion reversal ≥20% was more frequently observed in the IVT plus tirofiban group (adjusted odds ratio, 8.58; 95% CI, 2.09–35.28; p=0.001).
### Chart: IVT plus tirofiban
| Category | |
|---|---|
| Lesion reversal | 19.0 |
| No lesion reversal | 11.0 |
### Chart: IVT alone
| Category | Number |
|---|---|
| Lesion reversal | 6.0 |
| No lesion reversal | 22.0 |
CONCLUSIONS
IVT plus T improves radiological outcomes and may be a promising therapeutic option in patients with ACI/PPI, without increasing the risk of bleeding compared to IVT alone.
